# Development of the pediatric daily ulcerative colitis signs and symptoms scale (DUCS): qualitative research findings

**DOI:** 10.1186/s13104-017-2814-3

**Published:** 2017-09-25

**Authors:** Emuella Flood, Debra G. Silberg, Beverly Romero, Kathleen Beusterien, M. Haim Erder, Carmen Cuffari

**Affiliations:** 1 ICON plc, 820 W Diamond Ave, Ste 100, Gaithersburg, MD 20878 USA; 2 Shire, Wayne, PA USA; 3 ORS Health, Washington, DC USA; 4 The Johns Hopkins University School of Medicine, Baltimore, MD USA

**Keywords:** Patient-reported outcome, Observer-reported outcome, Diary, Content validation

## Abstract

**Background:**

The purpose of this study is to develop patient-reported (PRO) and observer-reported (ObsRO) outcome measures of ulcerative colitis (UC) signs/symptoms in children aged 5–17 with mild/moderate UC. The daily ulcerative colitis signs and symptoms scale (DUCS) was developed in two phases. Phase I involved concept elicitation interviews with patients and healthcare providers, review of website posts and item generation. Phase II involved cognitive debriefing and assessment of usability and feasibility of the eDiaries. Participants were recruited from five US clinical sites, a research recruitment agency, and internet advertising. Thematic and content analysis was performed to identify concepts from Phase I. The Phase II cognitive debriefing interviews were analyzed iteratively to identify problems with clarity and relevance of eDiary content. The US Food and Drug Administration (FDA) also reviewed and provided feedback on the eDiaries.

**Results:**

Phase I included 32 participants (22 remission; 10 active disease). Phase II included 38 participants (22 remission; 16 active disease). A core set of seven signs and symptoms emerged that were reported by at least 30% of the patients interviewed: abdominal pain, blood in stool, frequent stools, diarrhea, stool urgency, nighttime stools, and tiredness. Participant input influenced changes such as refinement of item wording, revision of graphics, and selection of response scales. Revisions suggested by FDA included simplifying the response scale and adding questions to capture symptoms during sleeping hours.

**Conclusions:**

The findings of instrument development suggest that the DUCS PRO and ObsRO eDiaries are content-valid instruments for capturing the daily signs and symptoms of pediatric patients with mild to moderate UC in a clinical trial setting.

## Background

Ulcerative colitis (UC) is a type of inflammatory bowel disease (IBD) characterized by inflammation of the large intestine and periods of disease exacerbation and remission [1]. IBD is diagnosed in childhood in up to 25% of patients, typically through endoscopy and tissue histology [2, 3]. Patients diagnosed with UC in childhood frequently have extensive disease with more acute and severe exacerbations versus those diagnosed as adults [2, 3]. Diarrhea, rectal bleeding, and abdominal pain are the hallmark signs and symptoms of UC. Treatment for pediatric UC is individualized depending on disease severity and may involve diet modification and medications, such as corticosteroids, 5-aminosalicylates, immunomodulators, and biologic therapies [1, 4].

Endoscopy provides an objective indicator of disease status and progression; however, endoscopy alone cannot provide the full picture of an individual’s disease experience. Assessment of symptoms provides important information on disease status and the overall benefit of treatment. Symptoms are known and best reported by patients themselves [5]. As such, the use of patient-reported outcomes (PROs) or observer-reported outcomes (ObsROs), for young children who are unable to self-report, is vital for accurately measuring treatment benefit in the pediatric clinical trial setting [6]. Guidelines for the development of PROs for use in clinical trials have been established by the US Food and Drug Administration (FDA) through the 2009 PRO Guidance [5]. The FDA guidelines place particular emphasis on the need for content validity based on direct research with patients, typically through interviews or focus groups. The International Society for Pharmacoeconomics and Outcomes Research (ISPOR) PRO good research practices for the assessment of children and adolescents task force has outlined good research practices specific to the development of PROs for children [6].

Based on a focused review of the literature, no pediatric UC PRO symptom measures were identified that would be fit for purpose and meet FDA and ISPOR task force guidelines with respect to instrument development and validation requirements. Only one PRO measure, the IMPACT-III, was identified as having been designed specifically for pediatric use in IBD [7]. The IMPACT-III is a patient-completed, health-related quality-of-life measure designed for children and adolescents aged 9 years and older with IBD; however, it does not have an observer-reported format and cannot capture the experience of younger children. Additionally, the IMPACT-III has a 2-week recall period and does not capture symptoms on a daily basis. It is also missing key signs and symptoms, such as blood and urgency. Another instrument, the Pediatric Ulcerative Colitis Activity Index (PUCAI) [3], is designed to assess disease activity in pediatric UC and includes symptom measurement; however, it is intended to be completed by the clinician rather than by the patient. The goal of this study was to develop a valid patient-reported (PRO) and observer-reported (ObsRO) measure, in the form of an electronic diary, to assess UC signs and symptoms in children with mild to moderate UC, aged 5–17 years inclusive.

## Methods

The electronic daily ulcerative colitis signs and symptoms scale (DUCS) was developed in two phases. Phase I involved open-ended concept elicitation interviews with children aged 8–17 years with mild to moderate UC, interviews with healthcare providers, and a review of UC-focused internet blogs. In Phase II, a cognitive debriefing, usability and feasibility assessment was performed on the draft electronic PRO and ObsRO diaries among patients with UC aged 8–17 years and caregivers of children with UC aged 5–10 years, respectively. Participants in both phases were recruited through five clinical sites in the United States (The Children’s Hospital, Aurora, CO; Cohen Children’s Medical Center, Lake Success, NY; Duke University, Durham, NC; Morristown Children’s Atlantic Center for Research, Morristown, NJ; The Johns Hopkins Children Medical Center, Baltimore, MD), as well as through a market research recruitment agency, and/or advertising via UC-focused websites. The study protocols for both Phase I and II were approved by local site-specific institutional review boards (IRBs), as well as by a commercial IRB (MaGil IRB; Rockville, MD). Written informed consent was received from the caregiver of each child participant, as well as from each caregiver participant prior to the start of the interview. Written assent was received from each child participant aged 12 years or older.

### Eligibility

Patient eligibility criteria were similar for both Phase I and Phase II. To be included in the study, a child had to be between the ages of 5 and 17 years, inclusive, with a documented history of UC confirmed by endoscopy. Children aged 8–17 participated in the concept elicitation and cognitive debriefing interviews. Caregiver participants had to be the caregiver of a child aged 5–10 years with confirmed UC. As part of the screening process, site coordinators or study staff administered the PUCAI as the assessment of disease severity; this was scored according to the developers’ guidelines [3]. Patients had to either have mild-to-moderate active disease or be in remission, based on their PUCAI score and elapsed time since their last flare. Active disease was defined as experiencing a flare at the time of the interview or having experienced a flare within the past 2 weeks with a PUCAI score of 10–64 at the time of the current or most recent flare. Patients in remission were defined as having experienced their most recent flare more than 6 weeks prior to screening with a PUCAI score < 10 at the time of the screening. Adequate written and oral fluency in English was required for both caregiver and child. Patients were excluded if they had severe UC as defined by a PUCAI score ≥ 65, proctitis only, or moderate or severe renal or hepatic impairment.

Physicians and nurses experienced in treating pediatric UC were interviewed prior to conducting the patient interviews. The goals of these interviews were to help inform the patient and caregiver interview guides, to gain an understanding of the disease from a clinician’s perspective, and to have the clinicians indicate the language their patients use to describe their signs/symptoms.

Supplementary data were also gathered through an analysis of patient and caregiver posts on a UC-focused website. These data provided additional confirmation of signs and symptoms identified during patient and caregiver interviews, as well as insight from caregivers of younger children. Blog posts were selected for review based on the following criteria: (1) the post had to specifically state that the child had been diagnosed with UC and (2) the reported age of the child had to be between 5 and 17 years. Posts were excluded if they stated that: (1) the patient had Crohn’s disease or (2) the patient had severe disease or a factor strongly indicating severe disease (e.g., the patient had had surgery for their UC).

### Study procedures

#### Phase I: concept elicitation

Open-ended concept elicitation interviews with patients were conducted between April and July 2012. The interviews were conducted in-person by research staff employed at each clinical site. Interviewers received training in qualitative interviewing. Participants recruited outside of clinical sites were interviewed by telephone by members of the study research staff experienced in qualitative interviewing. The interviews were conducted using semi-structured interview guides consisting of a series of open-ended questions that asked participants about their experience with UC signs and symptoms and daily life impacts. The interviews were designed to elicit spontaneous reports of signs and symptoms first, followed by probes on specific signs and symptoms. All patients, both active and in remission, were asked how they were currently feeling and were asked to describe the frequency, duration, severity, and impact of all signs and symptoms that they mentioned. Given the challenge of recruiting patients with active disease, those in remission were asked to describe their most recent flare. The goal of these questions was to help confirm data on signs and symptoms of active disease, including the language children use to describe their signs and symptoms.

Healthcare providers experienced in treating pediatric UC participated in hour-long, open-ended interviews conducted by trained research staff. Healthcare providers were asked to describe both the symptoms of pediatric UC and the specific words that patients and their parents use when describing UC signs/symptoms.

All interviews were digitally audio-recorded and transcribed. Participants were remunerated ($100 for caregivers and $25 for children) for their time to participate in the interview.

In addition, posts on a website devoted to UC were reviewed in order to provide additional information on how children and their caregivers describe pediatric UC symptoms. Posts dated between October 5, 2010 and June 20, 2012, written by either children with UC or their parents, were examined.

Draft PRO and ObsRO diaries were developed based on the signs and symptoms that patients and healthcare providers most commonly reported and that patients indicated were important. A draft conceptual framework was developed for each diary version (PRO and ObsRO), and items were generated to capture the severity of each sign and symptom selected for inclusion in the diary. Electronic versions of both the PRO and ObsRO diaries were programmed for use on handheld devices in preparation for the cognitive debriefing phase.

#### Phase II: cognitive debriefing

In cognitive debriefing interviews, children with UC aged 8–17 years provided feedback on the PRO version of the diary, and caregivers of children aged 6–10 years provided feedback on the ObsRO version. While it was attempted to also recruit caregivers of 5-year-olds, none were interviewed. For both caregivers and patients, the cognitive debriefing study included two interviews. The first was in-person to examine the clarity, relevance, and comprehensiveness of the diary content, including appropriateness of language and graphics across the full age range, along with the usability of the electronic device. To test usability, participants were asked questions related to their ability to use the device, see all of the questions/pictures clearly on each screen, select answers using the device, and navigate the screens on the device. The interview began with a brief concept elicitation on signs and symptoms, followed by an exploration of the meaning and interpretation of the diary content. Patients were also asked to provide bothersome ratings and importance rankings for their signs and symptoms. To test feasibility of completing the diary on a daily basis, participants then took the diary home and completed it each night for at least three nights.

The second interview was conducted by telephone between 3 and 7 days after the first interview to further examine the adequacy of the response scales, as well as the feasibility of completing the diary at home on a daily basis. The second interview lasted approximately 30 min and was focused on issues related to responding to the diary questions, recall, completing the diary at home, and usability (e.g., time needed to complete diary, forgetting to complete diary, needing to ask a parent for help in completing diary, and item clarity) of the electronic device. All interviews were audio-recorded with the participant’s permission and subsequently transcribed. Participants were remunerated ($100 for caregivers and $25 for children) for their time to participate in the interview.

### Analysis

A thematic and content analysis approach was used to identify concepts and to summarize and evaluate the data from the concept elicitation interviews with patients. Specifically, each transcript was reviewed to identify and enumerate themes representing signs and symptoms of UC. Assessment of information saturation, the point at which no new key themes are being identified with each successive interview, was assessed based on the number of new signs or symptoms being identified. Sign and symptom data reported by patients in remission describing a flare were compared with those reported by active patients describing their current symptoms. MAXQDA (version 10; VERBI GmbH; Berlin, Germany), a qualitative analysis software program, was used to help organize and categorize the data.

The Phase II cognitive debriefing interviews were conducted in small batches to allow for revisions to the diary between sets of interviews based on patient and caregiver feedback. Specifically, the cognitive debriefing interviews were analyzed iteratively with a focus on any instructions, items, or response options that were problematic with respect to their interpretability or relevance. Feedback from the FDA was sought at multiple time points during the instrument development process. As such, revisions to the DUCS were made based on feedback from cognitive debriefing interviews, as well as FDA feedback.

## Results

The demographic and clinical characteristics of the 32 patients interviewed in Phase I, which included 22 patients in remission and 10 patients with active disease, are provided in Table 1. Recruitment for Phase I took place between April and July 2012. The mean age was 14 years and about half were female. The characteristics of the patients and caregivers interviewed in Phase II are reported in Tables 1 and 2, respectively. The PRO cognitive debriefing sample consisted of 38 participants (22 females and 16 males), aged 8–17 years, with two of these participants testing different versions of the diary, for a total of 40 completed interview sets. Given the recommended cut-off scores for the PUCAI, the numbers of children in the remission, mild, and moderate disease groups were 22, 12, and 6, respectively. Participants were well-distributed across the United States, with the Northeastern, Mid-Atlantic, Midwestern, Western, and Southern regions represented. Caregivers of seven children, ages 6–10, participated in the cognitive debriefing of the ObsRO version. Participants for Phase II were recruited between November 2012 and August 2013.

**Table 1:** Patient characteristics: phase I and II

Patient characteristic	Total patients	Remission patients	Active patients
Phase I	n = 32	n = 22	n = 10
Age, y, mean ± SD	13.8 ± 2.6	14 ± 2.7	13.4 ± 2.6
Range	8–17	8–17	9–17
Gender, n (%)			
Female	18 (56)	12 (55)	6 (60)
Male	14 (44)	10 (45)	4 (40)
Race/ethnicity, n (%)			
Caucasian	26 (81)	17 (77)	9 (90)
African American	3 (9)	3 (14)	–
Asian	1 (3)	1 (5)	–
Latino/a	2 (6)	1 (5)	1 (10)
PUCAI total score, n (%)			
0	19 (59)	19 (86)	–
5	3 (9)	3 (14)	–
10	1 (3)	–	1 (10)
15	1 (3)	–	1 (10)
20	4 (13)	–	4 (40)
35	1 (3)	–	1 (10)
45	2 (6)	–	2 (20)
55	1 (3)	–	1 (10)

Patient characteristic	Total patients	Remission patients	Active patients
Phase II	n = 38^a^	n = 22^b^	n = 18^b^
Age, y, mean ± SD	12.84 ± 2.38	11.91 ± 2.11	14.06 ± 2.39
Median	13	12	14
Range	8–17	8–16	10–17
Gender, n (%)			
Female	22 (58)	12 (55)	12 (67)
Male	16 (42)	10 (45)	6 (33)
PUCAI total score, n (%)	n = 40^a^	n = 22^b^	n = 18^b^
Average score, mean ± SD	12.25 ± 14.36	1.81 ± 2.46	25.00 ± 12.37
Range	0–45	0–5	10–45
0	14 (35)	14 (64)	–
5	8 (20)	8 (36)	–
10	4 (10)	–	4 (22)
15	2 (5)	–	2 (11)
20	2 (5)	–	2 (11)
25	4 (10)	–	4 (22)
35	2 (5)	–	2 (11)
40	2 (5)	–	2 (11)
45	2 (5)	–	2 (11)

*PUCAI* Pediatric Ulcerative Colitis Activity Index, *SD* standard deviation, *UC* ulcerative colitis

^a^Two participants completed two sets of interviews, using two different diary versions. Both had different PUCAI scores at the time of each interview. Scores for all participants were calculated at the time of the first interview of each interview set

^b^Both participants who completed two sets of interviews changed disease status (i.e., remission vs active) between the first and second interview. For these participants, their characteristics are counted once in each disease status category

**Table 2:** Phase II caregiver/child (≤ 10 years) characteristics

Characteristics	Total caregivers (n = 7)^a^	Children in remission (n = 5)^b^	Children with active UC (n = 3)^b^
Caregiver’s age, y, mean ± SD	40.29 ± 5.79	44.00 ± 5.61	37.33 ± 6.11
Median	40	44	36
Range	32–49	37–50	32–44
Child’s age, y, mean ± SD	8.29 ± 1.70	9.20 ± 0.84	7.33 ± 2.31
Median	9	9	6
Range	6–10	8–10	6–10
Caregiver’s gender, n (%)			
Female	7 (100)	5 (100)	3 (100)
Male	0 (0)	0 (0)	0 (0)
Child’s gender, n (%)			
Female	4 (57)	4 (80)	1 (33)
Male	3 (43)	1 (20)	2 (67)
Child’s PUCAI total score as reported by caregiver, n (%)	(n = 8)^a^	(n = 5)^b^	(n = 3)^b^
Average score, mean ± SD	14.38 ± 14.2	2.00 ± 2.74	35.00 ± 10.00
Range	0–45	0–5	25–45
0	3 (38)	3 (60)	–
5	2 (25)	2 (40)	–
25	1 (13)	–	1 (33)
35	1 (13)	–	1 (33)
45	1 (13)	–	1 (33)

*PUCAI* Pediatric Ulcerative Colitis Activity Index, *SD* standard deviation, *UC* ulcerative colitis

^a^One participant completed two sets of interviews, using two different diary versions. The child had different PUCAI scores at the time of each interview. Scores for all participants were calculated at the time of the first interview of each interview set

^b^The participant who completed two sets of interviews changed disease status (i.e., remission vs active) between the first and second interview. For this participant, characteristics are counted once in each disease status category

A total of eight healthcare professionals (five pediatric gastroenterologists, and three clinical research coordinators including a pediatric nurse specializing in pediatric gastroenterology research) were also interviewed.

With respect to the UC-website post review, a total of 22 parents and four adolescents posted about UC impacts over the time period examined. Parents reported about their children in the age groups 5–7 years (n = 10), 8–12 years (n = 3), and 13–17 years (n = 9); the four children who posted were aged 13–17 years. The sample of all children who either posted or were described in the posts was 46% female.

### Concept elicitation findings

**Fig. 1:**
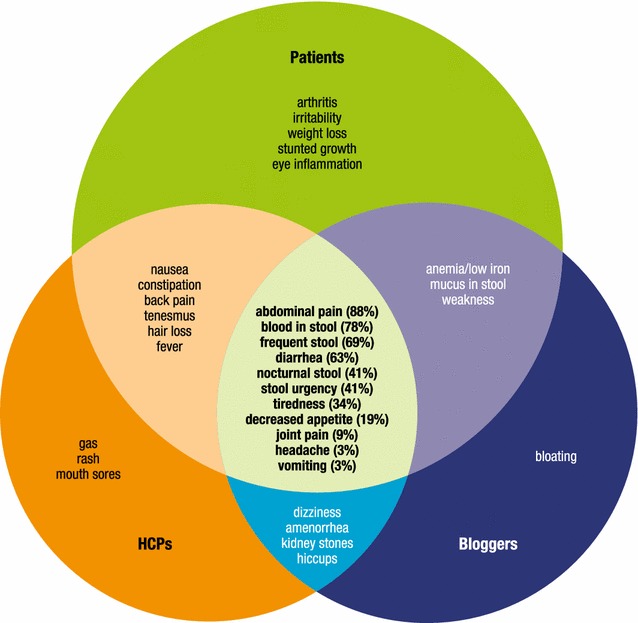
Signs and symptoms identified in concept elicitation. *HCP* health care professional

The signs and symptoms identified in the Phase I concept elicitation with the patients, healthcare providers, and website posts are presented in Fig. 1. A core set of seven signs and symptoms emerged that were reported by at least 30% of the patients interviewed and considered important to patients: abdominal pain (expressed as stomach pain, stomachache, or cramps), blood in stool, frequent stools, diarrhea, stool urgency, nighttime stools, and tiredness. Most patients in remission appeared to be able to easily recall the symptoms they experienced during their last flare, and the flare symptoms reported by these patients were consistent with those reported by the patients in a flare, with a similar pattern of frequency. Symptom experience and descriptions were generally similar across ages. Patients reported that the core signs and symptoms generally change from day to day and/or throughout the day, and that they are bothersome, interrupting regular activities, particularly school. Generally, patients reported not experiencing symptoms during remission; however, a small number of patients in remission did report experiencing symptoms within the past week, suggesting that not all patients in remission are symptom-free. Information saturation was achieved in identifying key signs or symptoms of pediatric UC.

The seven core signs and symptoms most frequently identified by patients were also identified by the health care professionals and in the parent/patient website posts. In addition, these were also reported by the patients participating in Phase II during the initial concept elicitation portion of the interview. The patients in Phase II did report additional signs/symptoms that had not been reported by patients in Phase I, but none of these additional signs/symptoms was identified by more than two of the 38 patients interviewed in Phase II. In response to the questions about sign/symptom ranking and bothersome ratings (0 = not at all bothersome; 10 = very bothersome) for the seven core UC signs/symptoms, the Phase II patients rated “blood in stool,” stomach pain,” and “rushing to the bathroom” as the most bothersome, with a mean rating of 6.5 out of 10 for each. However, substantial variability was found within the ratings of each sign/symptom, and all seven signs and symptoms were rated 9 or 10 (“very bothersome”) at least once.

### Item generation

**Fig. 2:**
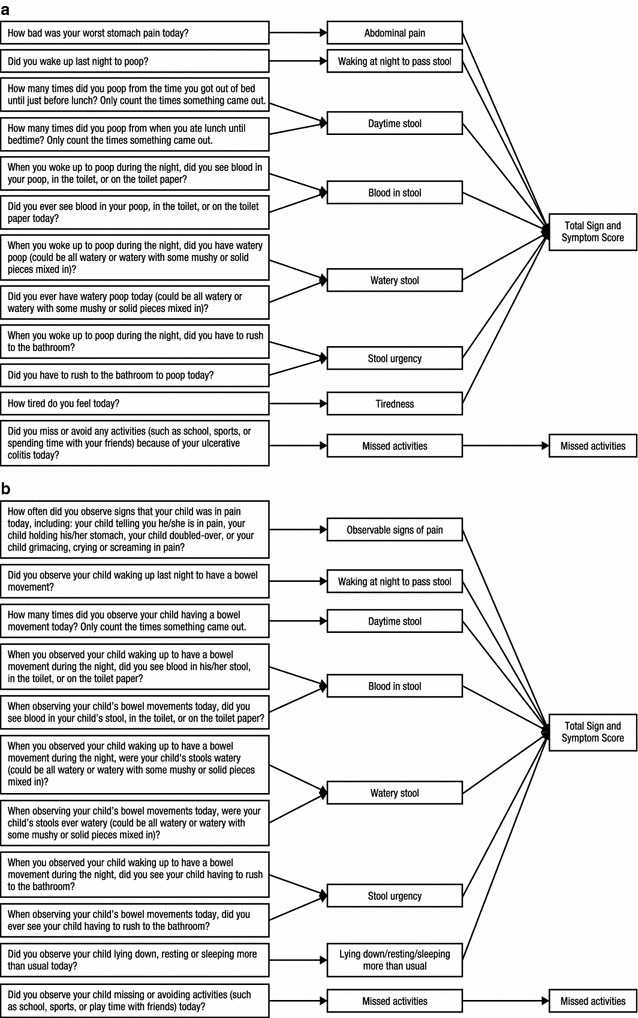
**a** Conceptual framework for patient-reported version of the DUCS. **b** Conceptual framework for observer-completed version of the DUCS. *DUCS* daily ulcerative colitis signs and symptoms scale

PRO and ObsRO electronic versions of the DUCS diary (eDiary) were developed focusing on the seven core signs/symptoms identified in concept elicitation. The PRO version was developed for the full age range of 8- to 17-year-old children with UC, either with active disease or in remission. Diary wording for the PRO was carefully selected to be age appropriate, understood by children as young as age 8 years, and reflective of patient descriptions reported in the concept elicitation interviews. While some children used clinical terminology to describe their symptoms (e.g., “stools” or “bowel movements”), less clinical terminology was used in the diary to ensure readability and comprehension. Graphics using a duck character were incorporated into the diary to help with overall child engagement and comfort in reporting on bowel-related symptoms. The ObsRO was limited to UC-related signs that could be observed by the parent/caregiver. Figures 2a and b show the conceptual framework for the PRO and ObsRO versions of the DUCS, respectively.

### Cognitive debriefing findings

The eDiary underwent a total of four revisions during the cognitive debriefing process. Revision 1 occurred after 23 child and four caregiver interviews had been completed. Revision 2 occurred after an additional three child interviews had been completed and after receiving comments from the FDA on the original version. Prior to testing Revision 2, additional feedback was received from the FDA, and therefore Revision 2 was not tested with patients. Revision 3 incorporated the additional feedback from the FDA and was tested with 14 children and four caregivers. Revision 4 incorporated final feedback and was considered the final version ready for psychometric testing.

Participant input influenced changes such as refinement of item wording for clarity, the revision of graphics, and the selection of the optimal pain scale. Revisions suggested by the FDA included simplifying the response scale by moving from a 5-point verbal rating scale to a dichotomous yes/no response for the questions on urgency, blood, and watery stool, as well as the addition of questions to capture these symptoms during sleeping hours.

The eDiary’s usability was also assessed, and both child and adult participants generally found the device easy to use and navigate. Some caregivers reported experiencing minor technical problems with the device, including difficulty with the alarms and PIN codes, which were recommended to be addressed prior to trial implementation.

## Discussion

To our knowledge, the DUCS is the first measure focusing on daily report of patient-reported signs and symptoms of UC in pediatrics, and thus fills an important need in pediatric UC research. Both the PRO and ObsRO versions of the DUCS are brief electronic tools that are easy to complete and capture the key UC signs and symptoms on a daily basis, which is particularly useful for a condition such as this with high variability in signs and symptoms occurring over a short period of time. Pending psychometric evaluation, the DUCS may be useful in clinical studies assessing outcomes in the pediatric UC population.

The FDA defines treatment benefit according to how a patient “feels, functions or survives” [5]. Thus, understanding the impact of a treatment on how patients feel is critical to drug development and regulatory approval. Only patients know how they feel, and the best way to capture this information is to ask patients directly. Evidence in the pediatric PRO literature suggests that children as young as 8 years old are able to reliably self-report on their health [6]. Consistent with this finding, our research showed that children down to age 8 are able to understand and respond to the DUCS. For children who are not able to reliably self-report, observer-reported measures based on parent observation are recommended.

The DUCS was developed to be a brief, simple, targeted, child-friendly, daily electronic measure of the core signs and symptoms reported by pediatric patients with mild to moderate UC. The FDA PRO guidance states that “when using multi-item instruments, it is important that all items be relevant to most of the patients in the clinical trial” (page 13) [5]. As such, the diary targets the most common and important symptoms reported by patients and confirmed by healthcare providers. Given the reported daily variability of symptoms, the diary is intended to be completed electronically on a daily basis to capture the variability, avoid recall bias, and maximize data accuracy.

The content of the DUCS is consistent with that of adult measures such as the Mayo, which assesses stool frequency and rectal bleeding, as well as the clinician-reported measure, the PUCAI, which also assesses stool frequency and rectal bleeding, in addition to abdominal pain, stool consistency, and nocturnal stools. With the exception of gas and nausea, which were not selected for inclusion in the DUCS due to their low frequency of mention, the DUCS also assesses the signs and symptoms covered by the IMPACT-III (e.g., stomach pain, diarrhea, and blood in stool). However, the DUCS has a 24-h recall period (compared to 2 weeks for the IMPACT-III and 2 days for the PUCAI) and also breaks down the day when asking about sign/symptom experience to help the child better remember the occurrence of the symptoms. It also assesses signs and symptoms not in the PUCAI and Mayo (i.e., urgency and tiredness) or IMPACT-III (i.e., stool frequency, blood in stool, urgency, and nocturnal stools).

Both the DUCS PRO and ObsRO were well-received by patients and caregivers in the cognitive debriefing interview phase of this study. With each round of revision, based on patient and caregiver input along with feedback from the FDA, the diaries were refined to improve patient understanding and item relevancy, resulting in a comprehensive daily diary for patients and caregivers. In terms of usability, both caregivers and patients found the diaries easy to use, understand, and navigate, as well as quick to complete. Some participants experienced technical problems related to the device itself, which would need to be remedied before trial use.

The development of this measure was not without limitations. Due to recruitment challenges, few parents of children in the 5- to 7-year age range were able to be recruited, and all of them were parents of children 6 years of age. Therefore, the data supporting the content validity for the ObsRO in this age range are limited. Nevertheless, the ObsRO was tested by parents of children up to age 10 years, and the results support the clarity, relevance, and appropriateness of the ObsRO for younger children. One limitation of the website post data set was that for most patients, disease severity was not explicitly reported; therefore, this sample may not have been limited to patients with mild and moderate disease. Additionally, for many of the posts, disease status (active or remission) was difficult to determine. Hence, although the website post data provided support for the patient interview findings, the limitations of this data set precluded it from being used in a more instrumental way in the development of the diaries.

## Conclusions

The findings of instrument development suggest that the final versions of the DUCS PRO and ObsRO diaries are content-valid instruments for capturing the daily signs and symptoms of pediatric patients with mild to moderate UC in a clinical trial setting. The DUCS fills an important need and was created according to good practice guidelines for the development of PROs [5, 6]. Future research is planned to assess the psychometric properties of the instrument within a clinical trial.

## Abbreviations

DUCS: Daily Ulcerative Colitis Signs and Symptoms Scale
eDiary: electronic diary
FDA: Food and Drug Administration
IBD: inflammatory bowel disease
IRB: institutional review board
ISPOR: International Society for Pharmacoeconomics and Outcomes Research
ObsRO: observer-reported outcome
PRO: patient-reported outcome
PUCAI: Pediatric Ulcerative Colitis Activity Index
UC: ulcerative colitis
